# Positron Emission Tomography studies with [^11^C]PBR28 in the Healthy Rodent Brain: Validating SUV as an Outcome Measure of Neuroinflammation

**DOI:** 10.1371/journal.pone.0125917

**Published:** 2015-05-21

**Authors:** Miklós Tóth, Janine Doorduin, Jenny Häggkvist, Andrea Varrone, Nahid Amini, Christer Halldin, Balázs Gulyás

**Affiliations:** 1 Department of Clinical Neuroscience, Karolinska Institutet, Centrum for Psychiatry Research, Stockholm, Sweden; 2 Department of Nuclear Medicine and Molecular Imaging, University of Groningen, University Medical Center Groningen, Groningen, The Netherlands; 3 Imperial College—NTU, Lee Chian Kong School of Medicine, Nanyang Technological University, Singapore; Chiba University Center for Forensic Mental Health, JAPAN

## Abstract

Molecular imaging of the 18 kD Translocator protein (TSPO) with positron emission tomography (PET) is of great value for studying neuroinflammation in rodents longitudinally. Quantification of the TSPO in rodents is, however, quite challenging. There is no suitable reference region and the use of plasma-derived input is not an option for longitudinal studies. The aim of this study was therefore to evaluate the use of the standardized uptake value (SUV) as an outcome measure for TSPO imaging in rodent brain PET studies, using [^11^C]PBR28. In the first part of the study, healthy male Wistar rats (n = 4) were used to determine the correlation between the distribution volume (V_T_, calculated with Logan graphical analysis) and the SUV. In the second part, healthy male Wistar rats (n = 4) and healthy male C57BL/6J mice (n = 4), were used to determine the test-retest variability of the SUV, with a 7-day interval between measurements. Dynamic PET scans of 63 minutes were acquired with a nanoScan PET/MRI and nanoScan PET/CT. An MRI scan was made for anatomical reference with each measurement. The whole brain V_T_ of [^11^C]PBR28 in rats was 42.9 ± 1.7. A statistically significant correlation (r^2^ = 0.96; p < 0.01) was found between the V_T_ and the SUV. The test-retest variability in 8 brain region ranged from 8 to 20% in rats and from 7 to 23% in mice. The interclass correlation coefficient (ICC) was acceptable to excellent for rats, but poor to acceptable for mice. In conclusion: The SUV of [^11^C]PBR28 showed a high correlation with V_T_ as well as good test-retest variability. For future longitudinal small animal PET studies the SUV can thus be used to describe [^11^C]PBR28 uptake in healthy brain tissue. Based on the present observations, further studies are needed to explore the applicability of this approach in small animal disease models, with special regard to neuroinflammatory models.

## Introduction

Activation of microglia has been implicated in the pathophysiology of many neurological and psychiatric diseases [1,2]. Although the presence of activated microglia has been shown in these diseases, the cause, course and consequences of this microglia activation remain unknown. In order to understand the role and nature of microglia activation in brain disease processes, studies in small animals are of major importance. These studies make it possible to explore, among others, the events that trigger the process, the on-set and progression of microglia activation, differences in microglia phenotype and the mechanisms and efficacy of interventions aimed at reducing microglia activation, all of which are more difficult to study in a clinical setting. Small animal positron emission tomography (PET) is of great value for studying microglia activation in disease models in rodents, especially because the technique allows for non-invasive assessment of longitudinal changes.

Imaging of microglia activation with PET makes use of radiolabeled ligands of the 18 kD translocator protein (TSPO), known previously as the peripheral benzodiazepine receptor (PBR) [3]. The TSPO is a five transmembrane protein which is located in the outer mitochondrial membrane and plays a crucial role in cell physiology [4,5]. In normal brain tissue the expression of TSPO is low, but with the activation of microglia the TSPO expression increases [2]. The first radioligand that was widely used for imaging of microglia activation was [^11^C]PK11195 [6]. However, this radioligand has numerous limitation [7], including a high level of non-specific binding and a poor signal-to-noise ratio. Several other radioligands have been developed [1], of which [^11^C]PBR28 is a radioligand with high affinity for the TSPO and showing a good ratio between specific and non-specific binding [8]. [^11^C]PBR28 was found to have a higher specific binding than [^11^C]PK11195 and it has been used to show microglia activation in animal models [8,9] and in clinical studies [10,11]. [^11^C]PBR28 is thus a promising radioligand for imaging neuroinflammation in animal models of human neurological diseases using small animal PET.

Whereas small animal PET is the ideal tool for longitudinal monitoring of animal models, there are limitations when it comes to quantification of the PET data. For quantification of PET data of radioligands for the TSPO, including [^11^C]PBR28, a plasma derived input function is required which is obtained by measuring radioactivity in arterial blood. In small animals, like rats and mice, this is a rather difficult task. The arterial cannulation and the amount of blood samples generally require that the animal is terminated afterwards. The continuous loss of blood during the experiment is affecting the circulating blood volume and can, consequently, affect the calculation of the volume of distribution. Additionally, quantification cannot be done using reference tissue modeling, which does not require arterial sampling, because there is no suitable reference region [12]. An appropriate solution for longitudinal small animal studies would be to use the semi-quantitative standardized uptake value (SUV) as a measure of [^11^C]PBR28. The feasibility of using the SUV in rodents has not yet been investigated for [^11^C]PBR28 or any of the other TSPO ligands in either healthy animals or small animal disease models. Therefore, the aim of the present study was to evaluate the use of the SUV as the outcome measure in [^11^C]PBR28 small animal PET studies in healthy rats and mice.

## Materials and Methods

### Animals

Eight healthy male Wistar rats (363 ± 57 g) and four healthy male C57bl/6J mice (25.0 ± 0.5g) from Charles River Laboratories (Sulzfeld, Germany) were used for the PET measurements. The animals were housed in a thermo regulated (~22°C) and humidity-controlled facility under a 12 h/12 h light/dark cycle with access to food and water ad libitum.

All animal experiments were conducted according to the appropriate Swedish regulations with the approval of the Animal Research Ethics Committee of the Swedish Animal Welfare Agency (Northern Stockholm Region) and were performed according to the guidelines of Karolinska Institutet regarding working with experimental animals (Dnr.: N210/10).

### Study design

The study was divided in two parts. In the first part arterial sampling was performed in four rats to allow for quantification of [^11^C]PBR28 binding and comparison with the SUV. In the second part four rats and four mice were used to determine the test-retest variability of the SUV, with a 7-day interval between the scans.

### Radiochemistry

[^11^C]PBR28 (N-acetyl-N-(2-[^11^C]methoxybenzyl)-2-phenoxy-5-pyridinamine) was synthesized through partial modification of a previously described method [13]. In short, [^11^C]CH_4_ was released from the target and collected in a Porapak Q trap cooled with liquid nitrogen. Following collection the [^11^C]CH_4_ was released from the trap by heating with pressurized air and subsequently [^11^C]CH_4_ was mixed with vapors from iodine crystals at 60°C followed by a radical reaction occurred at 720°C. After the reaction, [^11^C]CH_3_I was collected in a Porapak Q trap at room temperature and the unreacted [^11^C]CH_4_ was recirculated for 3 min. [^11^C]CH_3_I was released from the Porapak Q trap by heating the trap using a home-produced oven to 180°C. [^11^C]methyl triflate was prepared by sweeping [^11^C]CH_3_I vapour through a heated glass column containing silver-triflate-impregnated graphitized carbon. The radiosynthesis of [^11^C]PBR28 was obtained by trapping [^11^C]methyl triflate at room temperature in a reaction vessel containing precursor desmethyl PBR28 (0.3–0.6mg, 0.9–1.8 μmol) and tetrabutylammonium hydroxide (1.0 M in MeOH, 2 μL) in acetone (300μL). After the reaction, the mixture was diluted with 500 μL of HPLC eluent before being injected to the HPLC system to purify [^11^C]PBR28. The final product was isolated and formulated in phosphate buffered saline (pH = 7.4) containing less than 10% ethanol. The radiochemical purity was > 95% and specific activity at time of injection was 673 ± 426 GBq/μmol (n = 8) in mice, and 2378 ± 5034 GBq/μmol (n = 8) in the rat. The precursor and standard were supplied by PharmaSynth AS, Tartu, Estonia.

### In vivo imaging with small animal PET

The animals were anesthetized with isoflurane (induction 4–5% mixed with oxygen and maintenance 1.5–2% mixed with air/oxygen (50/50)) at a flow rate of 1 L/min and a cannula was inserted into the tail vein for the injection of [^11^C]PBR28. After the cannulation, the rats were positioned into the nanoScan PET/MRI or nanoScan PET/CT (Mediso Ltd., Budapest, Hungary) [14,15] in a transaxial position with their heads in the centre of the field of view. A bolus injection of [^11^C]PBR28 was given via the canula in the tail vein at start of the PET measurement. The injection was followed by a saline flush. An emission scan of 63 minutes was acquired for all animals. The average injected radioactivity was 23 ± 5 MBq for the rats and 9.9 ± 1.6 MBq for the mice. The injected mass was 0.05 ± 0.03 μg (n = 8) in rats and 0.011 ± 0.011 μg (n = 8) in mice.

The PET data was acquired in list mode and was reconstructed into 25 timeframes (4x10 s, 4x20 s, 4x60 s, 7x180 s, 6x360 s) with MLEM reconstruction (20 iterations). Image data were not corrected for attenuation or scatter. For each animal a T1-weighted MRI scan (Isotropic FSE sequence, TR/TE: 2000/84.1) was made for anatomical reference.

### Arterial blood sampling

Arterial blood sampling was performed during the PET scan in the first group of four rats. In addition to the insertion of the cannula in the tail vein, another cannula was inserted into the femoral artery. For the rats, sixteen arterial blood samples of 0.1 ml were taken for measurement of radioactivity in plasma and blood using a gamma counter. Four samples of 0.5 ml blood were taken for radiometabolite analysis at 4, 20, 40 and 60 minutes after injection.

### Radiometabolite analysis

To determine the percentages of radioactivity in plasma that corresponded to unchanged [^11^C]PBR28 and its radioactive metabolites, reversed-phase HPLC was used. Plasma obtained after centrifugation of 0.5 ml blood at 2,000 g for 2–4 min was mixed with acetonitrile (0.35 ml) and centrifuged for 4 min. The acetonitrile extraction ratio was 93.5% ± 1.8% at 4 min, 87.8% ± 0.5% at 20 min, 85.2% ± 1.1% at 40 min and 78.9% ± 2.4% at 60 min. The supernatant was mixed with 2 ml of water and analysed using radio-HPLC. The radio-HPLC system used consisted of an interface module (D-7000; Hitachi: Tokyo, Japan), a L-7100 pump (Hitachi), an injector (model 7125, with a 5.0-mL loop; Rheodyne: Cotati, USA) equipped with a μ-Bondapak C18 column (300 x 7.8 mm, 10 mm; Waters: New England, USA), and an ultraviolet absorption detector (L-7400, 254 nm; Hitachi) in series with a 150TR; Packard (housed in a shield of 50 mm thick lead) equipped with a 550 μL flow cell. Acetonitrile (A) and ammonium formate (100 mM) (B) were used as the mobile phase at 6.0 mL/min, according to the following gradient: 0–4 min (A/B), 40:60 → 80:20 v/v; 4.1–6 min(A/B), 80:20 v/v; 8 min (A/B), 40:60 v/v. Peaks for radioactive compounds eluting from the column were integrated and their areas were expressed as a percentage of the sum of the areas of all detected radioactive compounds (decay-corrected to the time of injection on the HPLC).

### Data Analysis

The reconstructed dynamic PET measurements were manually aligned with the corresponding MRI scans. The MRI and PET scans were then normalized to the rat or mice brain MRI template available in PMOD (PMOD 3.2 Zurich, Switzerland). Eight regions of interest were defined using the available template in PMOD, i.e. the striatum, hippocampus, hypothalamus, midbrain, thalamus, cerebral cortex, brainstem and cerebellum. Time-activity curves were generated for each region of interest. For all animals the regional brain uptake values were expressed as SUV and were calculated for 57–63 minute after injection, i.e. the last time-frame. The time used for calculating the SUV was based on the tissue-to-plasma ratio obtained from the first part of the study. From 15–50 minutes the tissue-to-plasma ratio is declining, and the estimated equilibrium state is reached at 50–60 minutes after injection. However, it can be beneficial for future studies to use a shorter scan time, i.e. to allow for scanning of more animals per batch of [^11^C]PBR28 and to improve counting statistics. The SUV was therefore also calculated for the time-frame of 39–45 minute after injection, In both cases the SUV was calculated according to Eq 1, and it was assumed that 1 cm^3^ of brain tissue equals 1 g.

$$SUV=\frac{\text{tissue activity concentration}\;\left(\text{Bq}/\text{cm}3\right)}{\text{injected dose}\;\left(\text{Bq}\right)\;/\;\text{body weight}\;\left(\text{g}\right)}$$ (1)

For the first groups, the arterial samples and percentage of metabolites were used to determine the metabolite corrected plasma-input function. Two-tissue compartment modelling (2-TCM) and Logan graphical analysis [16] were used to calculate the volume of distribution (V_T_), using the metabolite corrected plasma curve as input. For the Logan analysis data was fitted from 21 minutes after injection (t* = 21 min).

For the test-retest study the test-retest variability was calculated using Eq 2.

$$TRV=\frac{\left|\text{test value}\;-\;\text{retest value}\right|}{\left(\text{test value}\;+\;\text{retest value}\right)\;/\;2}*100$$ (2)

Additionally the interclass correlation coefficient (ICC) was calculated as an index of the reliability of the test-retest measurement using Eq 3, where MS = mean sum of squares, BS = between-subjects, WS = within-subjects, and *df* = degrees of freedom. It combines the information of the difference between and within the test and retest scan. The ICC values were interpreted as follows: 0–0.2 indicated slight agreement, 0.3–0.4 indicated fair agreement, 0.5–0.6 indicated moderate agreement, 0.7–0.8 indicated substantial agreement, and >0.8 indicated almost perfect agreement between the test and retest measures [17].

$$ICC=\frac{\text{MSBS}-\text{MSWS}}{\text{MSBS}+\left(df\text{WS}*\text{MSWS}\right)}$$ (3)

### Statistical analysis

All data are expressed as mean ± standard deviation. Statistical analysis was performed using PASW for Windows, version 18.0. One-way ANOVA was used for the between group comparisons. For statistical analysis of the time-activity curves the general linear model repeated measure was used. Correlations were analyzed by using Pearsons’s correlation coefficient. Significance was reached when the p value was <0.05.

## Results

### Small animal PET of [^11^C]PBR28

**Fig 1:**
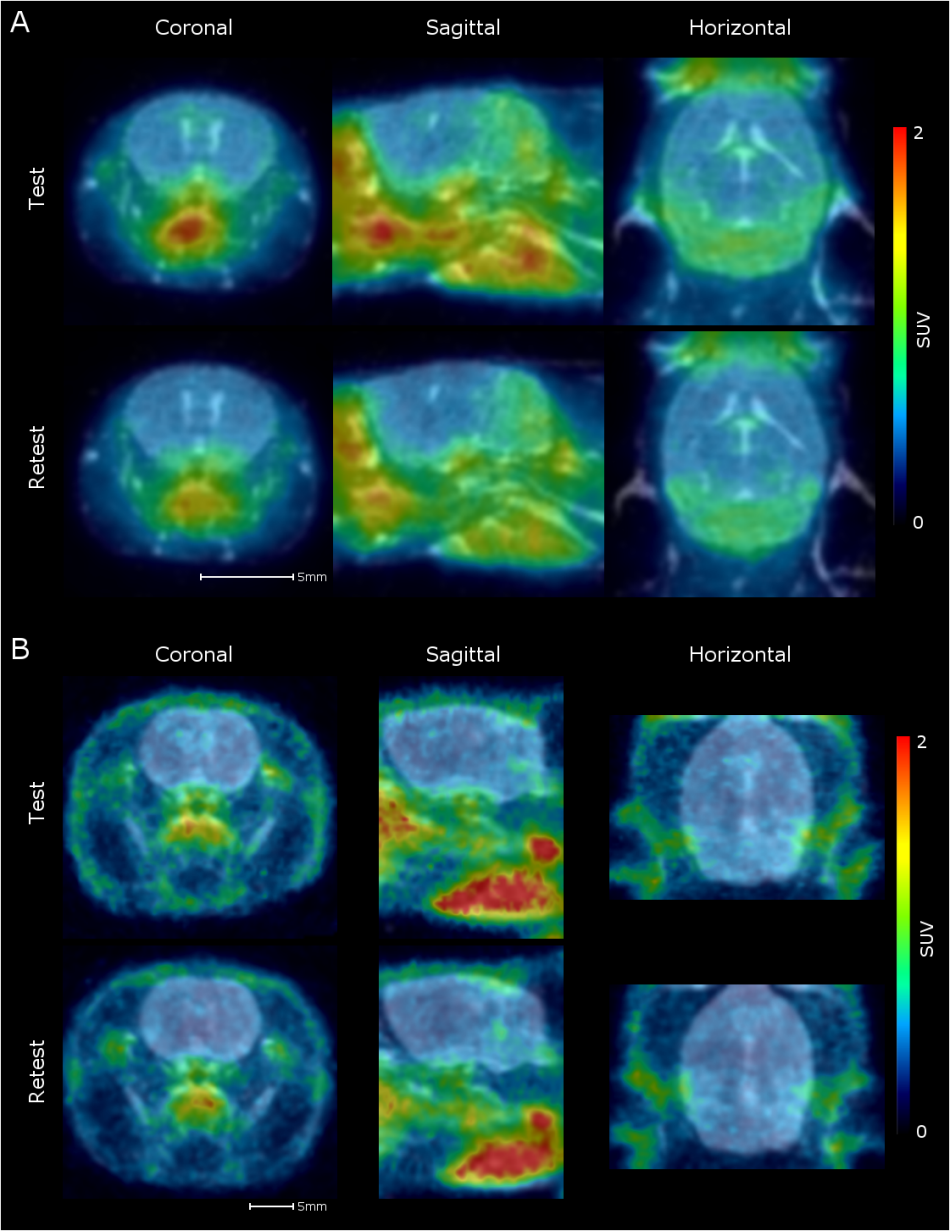
Representative PET images of a rat and a mouse of the test-retest part of the study, co-registered with the MRI scan. PET images of a representative rat and mouse are shown overlaying the individual MRI measurements.

**Fig 2:**
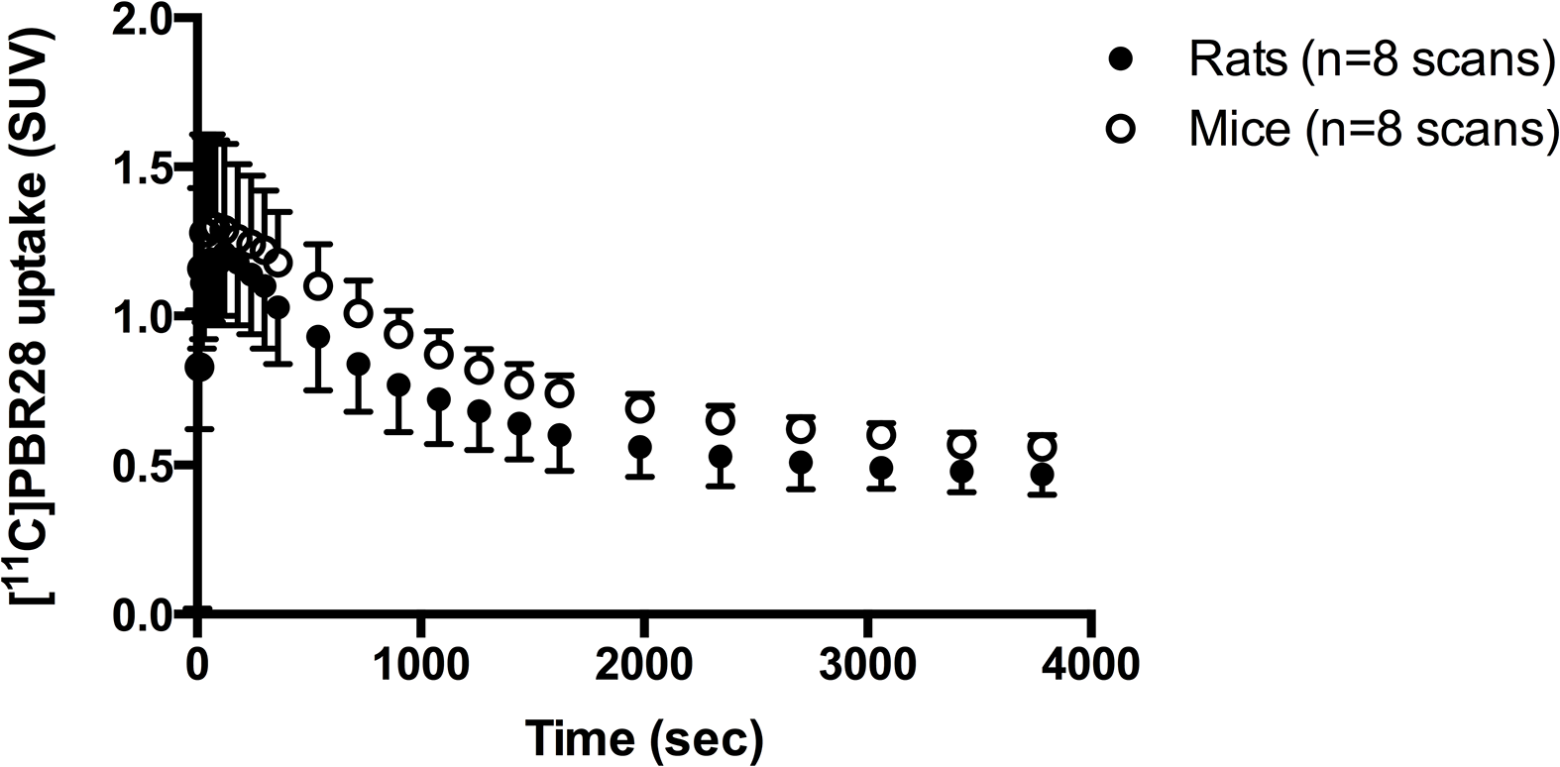
Total brain time-activity curves of [^11^C]PBR28 for rats and mice (SUV). The time-activity curves represent the average uptake of the eight scans that were performed in both four rats and mice for the test-retest part of study, in which no arterial sampling was performed.

Representative small animal PET images of a rat and mouse brain are displayed in Fig 1. The time-activity curve of the total brain in non-sampled rats and mice (Fig 2) show that the uptake (SUV) was maximum around 1 min after injection for both rats and mice. From around 40 minutes after injection [^11^C]PBR28 uptake remained relatively stable over time, although a small decrease could still be observed. When comparing the time-activity curves between rats and mice, [^11^C]PBR28 uptake seems to be higher in mice throughout the entire duration of the scan but this did not reach statistical significance (p = 0.057). However, a statistical significant difference in the interaction between time and the group was found (p > 0.001), showing that the change in [^11^C]PBR28 uptake over time was different for rats and mice.

**Fig 3:**
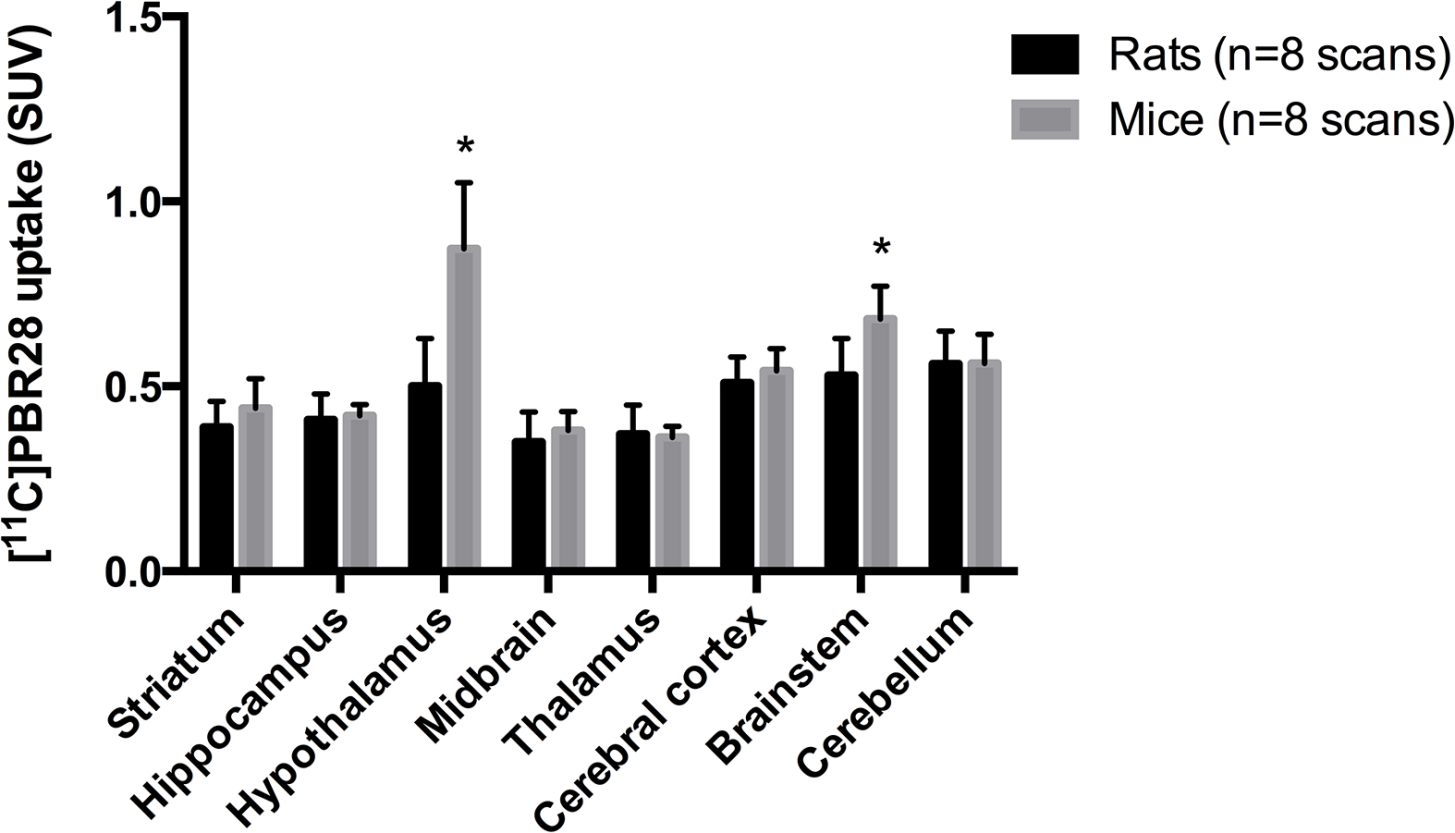
[^11^C]PBR28 uptake (SUV) in eight different brain regions at 63 minutes after injection, for both rats and mice. The SUV values represent the average of the eight scans that were performed in both four rats and mice for the test-retest part of study, in which no arterial sampling was performed. * p<0.05.

Regional differences in [^11^C]PBR28 uptake (SUV) were observed (Fig 3). In rats, the highest uptake was observed in the cerebellum, brainstem and cerebral cortex, followed by the hypothalamus. Highest uptake in mice was found in the hypothalamus, followed by the brainstem, cerebellum and cerebral cortex. For both rats and mice the lowest uptake was found in the striatum, hippocampus, midbrain and thalamus. When comparing [^11^C]PBR28 uptake between rats and mice, a statistically significant higher uptake was found in the hypothalamus (p < 0.001) and brainstem (p < 0.011) of mice.

### Kinetic modeling of [^11^C]PBR28 in rats

**Fig 4:**
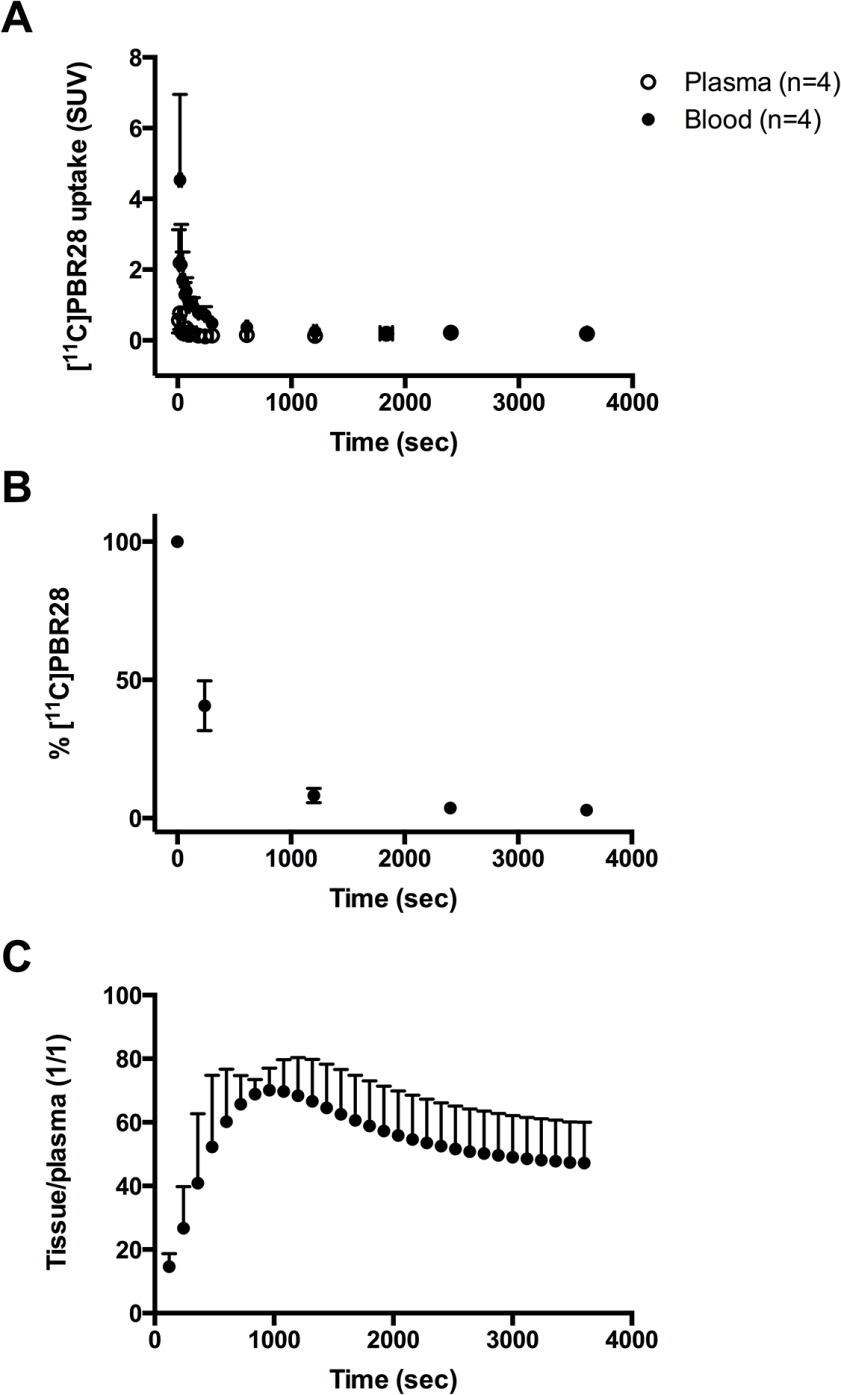
Blood and plasma time activity curves (A), the percentage of [^11^C]PBR28 in plasma (B) and the tissue-to-plasma ratio (C) in rats.

Arterial blood sampling was performed so that a metabolite-corrected plasma input curve could be generated. The blood and plasma time-activity curves (Fig 4A) show that uptake peaked around 20 seconds after injection of [^11^C]PBR28. Uptake in blood was found to be higher than in plasma. On average the plasma/blood ratio of radioactivity was 0.2 at 20 seconds after injection and gradually increased to a ratio of 1.0 at 60 minutes after injection.

Blood samples for metabolite analysis were taken at 3–4 time points: at 4, 20, 40 and 60 minutes (the 40 minute sample was only taken in one of the four rats) after injection of [^11^C]PBR28. [^11^C]PBR28 was rapidly metabolized in the rats (Fig 4B). At 4 minutes after injection 40.7 ± 9.0% of the parent compound was left, meaning that about 60% was metabolized. At 20 minute after injection only 8.2 ± 2.6% of the parent compound was left, which was reduced to 3.7% (n = 1) at 40 minutes after injection and to 2.9 ± 1.5% at 60 minutes after injection.

The curve of the percentage of parent compound over time showed a two-phase decay.

**Fig 5:**
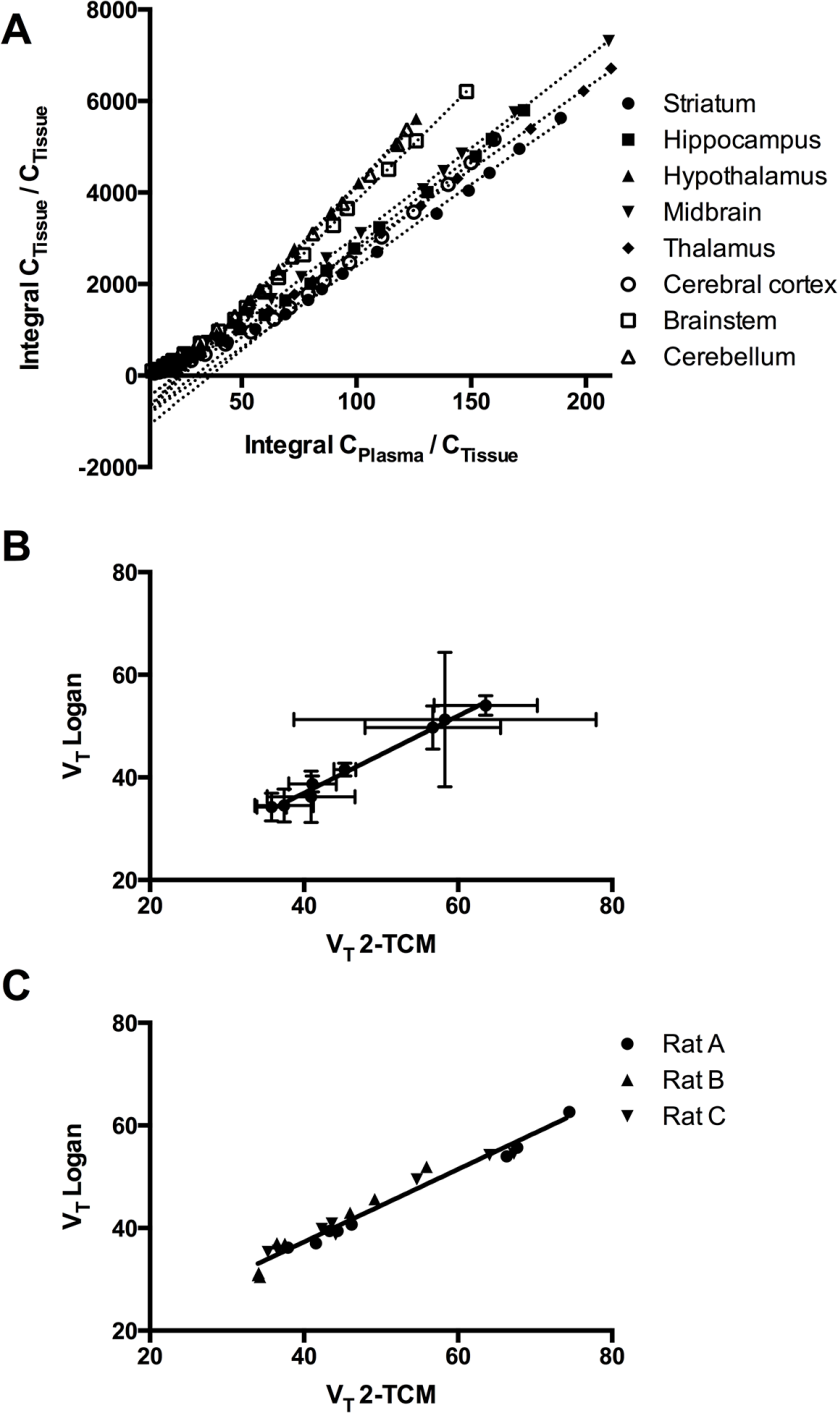
Correlation between V_T_ obtained with two-tissue compartment modeling (2-TCM) and Logan analysis. Logan plots (A) and the correlation between V_**T**_ of [^**11**^C]PBR28 as calculated using 2-TCM and Logan graphical analysis [16], for the averages of eight brain regions (B) and for the individual values for each rats for the eight brain regions (C).

2-TCM and Logan analysis (Fig 5) fitted the data for three of the four rats and one rat was therefore excluded from the data. For 2-TCM the tissue and blood/plasma data obtained for 60 minutes was used. 2-TCM with a shorter time (i.e. 30 and 40 minutes) was explored, but resulted in a higher variability in the V_T_ (data not shown).

The total brain V_T_ obtained from 2-TCM and Logan analysis were found to be, respectively, 47.3 and 43.1 on average. For the individual brain areas, the V_T_ ranged from 35.8 to 63.6 for 2-TCM and from 34.4 to 54.0 for Logan analysis (Table 1). When comparing the V_T_ values obtained from 2-TCM and Logan analysis for the eight different brain regions, a high correlation was found (r^2^ = 0.99). A similar high correlation was found when comparing all the values of the individual rats were taken for the eight brain regions (r^2^ = 0.90).

**Table 1:** V_T_ of [^11^C]PBR28 in various brain structures in rats calculated using two-tissue compartment modelling (2-TCM) and Logan graphical analysis [16].

	V_T_ 2-TCM (n = 3)			V_T_ Logan(n = 3)		
Striatum	37.4	±	3.8	34.5	±	3.2
Hippocampus	41.1	±	3.1	38.7	±	1.6
Hypothalamus	58.3	±	19.6	51.3	±	13.1
Midbrain	40.9	±	5.7	36.2	±	5.0
Thalamus	35.8	±	1.9	34.2	±	2.7
Cerebral cortex	45.3	±	1.4	41.5	±	1.3
Brainstem	56.7	±	8.8	49.7	±	4.2
Cerebellum	63.6	±	6.7	54.0	±	1.9

**Fig 6:**
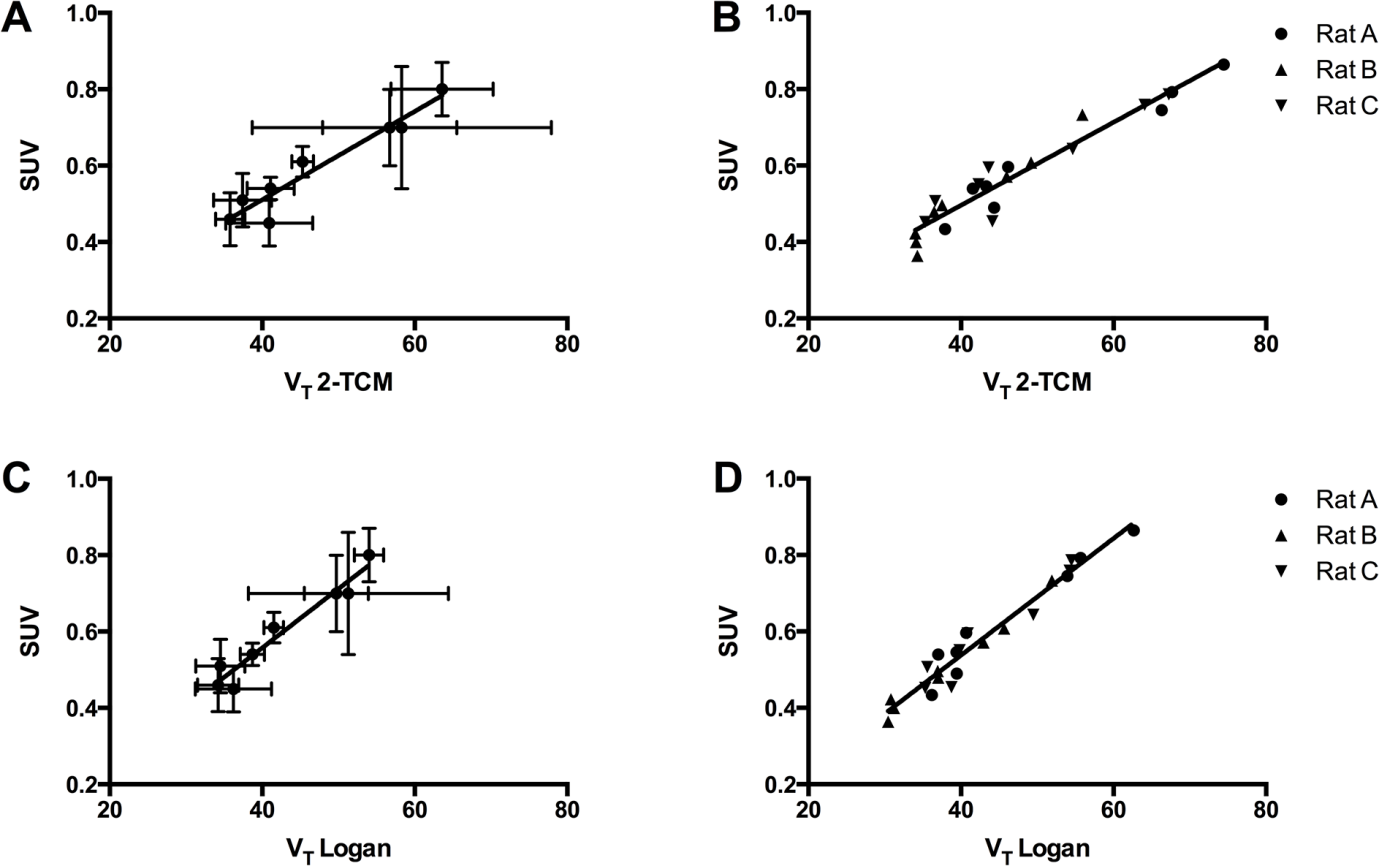
Correlation between V_T_ and SUV of [^11^C]PBR28 in rats. Correlation between VT of [11C]PBR28 as calculated using two-tissue compartment modeling (2-TCM) (A+B) and Logan graphical analysis (C+D) [16], and the SUV of [11C]PBR28, for the averages of eight brain regions (A+C) and for the individual values for each rats for the eight brain regions (B+D).

The SUV of the time-frame of 57–63 minutes was calculated for the sampled rats and it was correlated to the V_T_ (Fig 6). A high correlation between the average V_T_ for eight different brain regions and the average SUV was found; r^2^ = 0.92 for 2-TCM (p<0.001) and r^2^ = 0.94 for Logan analysis (p<0.001). The individual correlations for each rat ranged from 0.87–0.95 (slope of 0.0099 to 0.0160) for 2-TCM and from 0.91 to 0.98 for Logan analysis (slope of 0.0148 to 0.1540). Additionally, a high correlation between the V_T_ and the SUV was found when all the values of the individual rats were taken for the eight brain regions, instead of the average; r^2^ = 0.92 for 2-TCM (p<0.001) and r^2^ = 0.95 for Logan analysis (p<0.001).

For 2-TCM the individual correlations between V_T_ and SUV (n = 3 per correlation) ranged from 0.83 to 1.00 for six out of eight brain regions, with a slope of 0.0094 to 0.0145. For the Logan analysis the individual correlations for 6 out of 8 brain area ranged from 0.95 to 1.00, and the slopes of the relation ranged from 0.0127 to 0.0194. For both the 2-TCM and Logan analysis the thalamus (2-TCM, slope of 0.0064, r^2^ = 0.21; Logan analysis, slope of 0.0088, r^2^ = 0.75) and the cortex (2-TCM, slope of -0.0039, r^2^ = 0.15; Logan analysis, slope of -0.0011, r^2^ = 1.00) showed a deviant correlation.

When the SUV of the time-frame of 39–45 minutes was compared to the V_T_ obtained from 2-TCM and Logan analysis, similar correlation were found as for the time-frame of 57–63 minutes (data not shown). When comparing the average for the eight different regions, the rats individually or the values for all rats and the eight regions together, a high correlation between the SUV and V_T_ was found: r^2^ = 0.91 to 0.98 for 2-TCM (p<0.001) and r^2^ = 0.94 to 0.99 for Logan analysis (p<0.001). The individual correlations for six out of eight brain regions (excluding the thalamus and cortex because of the deviant correlation) ranged from 0.62 to 1.00 for 2-TCM and from 0.77 to 1.00 for Logan analysis.

### Test-retest variability of the SUV in mice and rats

The test-retest variability of the SUV of the time-frame of 57–63 minutes for the eight different brain regions in rats ranged from 7.7 ± 3.5 for the hippocampus to 20.4 ± 15.4 for the hypothalamus (Table 2). In mice, the test-retest variability ranged from 6.9 ± 4.7 for the hippocampus to 23.6 ± 15.6 for the striatum. In addition to the test-retest variability the interclass correlation coefficient (ICC) was calculated (Table 2). For rats the ICC was lowest for the brainstem (0.58) and highest for the hippocampus (0.88). The ICC for mice was found to be lower than for rats, and was lowest for the hypothalamus (0.01) and highest for the hippocampus (0.61). For the mouse striatum and cerebral cortex the ICC could not be determined due to violation of assumptions, i.e. a negative average covariance among the test and retest scan.

**Table 2:** Test-retest variability (TRV) and interclass correlation coefficient (ICC) of [^11^C]PBR28 for rats and mice that were scanned twice, with an interval of 7-days.

	Rats				Mice			
	TRV			ICC	TRV			ICC
Striatum	13.6	±	4.8	0.69	23.6	±	15.6	ND
Hippocampus	7.7	±	3.5	0.88	6.9	±	4.7	0.32
Hypothalamus	20.4	±	15.4	0.59	23.0	±	17.6	0.01
Midbrain	12.3	±	15.8	0.86	15.1	±	6.3	0.02
Thalamus	13.7	±	9.2	0.71	7.6	±	4.6	0.71
Cerebral cortex	16.7	±	9.2	0.81	13.4	±	11.6	ND
Brainstem	17.7	±	7.8	0.58	13.2	±	9.1	0.31
Cerebellum	11.5	±	7.4	0.72	13.7	±	6.4	0.29

For the SUV of the time-frame of 39–45 the test-retest variability was comparable to the time-frame of 57–63: for rats it ranged from 12.2 ± 7.9 to 22.0 ± 3.6 and for mice from 8.6 ± 6.9 to 19.6 ± 14.5. The ICC values for rats were found to be comparable to the time-frame of 57–63 (0.59 to 0.82), but for mice the values were found to be higher (0.28 to 0.63).

## Discussion

The present study have demonstrated that SUV is a reliable outcome measure for [^11^C]PBR28 in rats. The SUV showed a high and statistical significant correlation with the V_T_ obtained with 2-TCM and Logan analysis. The test-retest variability was similar for rats and mice, and showed a good reproducibility.

For practical reasons, the use of the SUV as an outcome measure in small animal studies using TSPO ligands is preferred over quantification, as it does not require blood sampling. Blood sampling in rodents limits performing longitudinal studies, as the blood sampling procedure generally requires termination of the rodent after the scan. Especially in studies on TSPO expression in neuroinflammation, there is a need to perform longitudinal experiments to assess disease progression and the efficacy of treatment. But to determine whether the SUV can be used as an outcome measure, it has to be compared to quantitative outcome measures, such as the volume of distribution (V_T_) or the binding potential (BP_ND_). For clinical studies with [^11^C]PBR28 the two-tissue compartment model is generally used for calculation of the V_T_ and BP_ND_. In a first study on TSPO quantification in rats with cerebral ischemia, Imaizumi *et al*. [9] showed that the V_T_ was well identified with the two-tissue compartment model. We therefore also selected the V_T_ as the outcome measure for quantification in the present study and calculated the V_T_ with the 2-TCM and Logan graphical analysis [16]. There was a high correlation between the V_T_’s obtained with 2-TCM and Logan analysis, showing the feasibility of both methods in rats. The values for V_T_ were found to be higher than those reported by Imaizumi *et al*. [9]. They reported a V_T_ value of 13.5–21.5 for the striatum at the non-ischemic side after a bolus injection of 6 minutes. Although the values for the striatum were found to differ between both studies, they are both higher than V_T_ values reported in human studies [18]. The high values for V_T_ are related to the low plasma availability of [^11^C]PBR28 at the estimated equilibrium. The low plasma availability and the assumed low binding to TSPO in healthy rats together suggest that the high V_T_ is mainly explained by high non-specific binding in brain tissue. This could hamper the detection of small increases in TSPO expression in disease models. Currently the number of small animal studies with [^11^C]PBR28 are limited and this therefore remains to be investigated.

The high correlation between the V_T_ and SUV in the present study is consistent with the findings of a high correlation between the V_T_ and SUV of [^11^C]PBR28 in baboons [19]. However, they found that the slope of the relation between V_T_ and SUV showed a high variation across different PET measurements. Additionally, the correlation decreased when the V_T_ increased as a consequence of LPS injection (i.e. inducing neuroinflammation) because the SUV remained static. In our study in rats we have found that the slope of the relation between V_T_ and SUV was consistent amongst the three rats and amongst brain regions. We have not included a disease model with increased TSPO expression in our study, but based on previous findings on the use of SUV for TSPO ligands it is expected that there will be an increase in the SUV in neuroinflammation [20] rather than the SUV remaining static as reported by Yoder *et al*. [19]. If the correlation with the V_T_ will be similarly high in a disease model as in control rats remains to be investigated.

For the comparison with the V_T_ and SUV it has to be taken into account that the SUV obtained from the rats of which blood samples were taken during the scan, can be affected by the large amount of blood (about 13%) taken for the determination of the blood- and plasma-input curves and radiometabolites. Indeed, the SUV of the sampled rats was about 30% higher (data not shown) than the SUV of the non-sampled rats from the test-retest study. A higher SUV in sampled rats is in contrast to what was expected. Because of the blood samples taken the amount of blood, and thus the amount of available [^11^C]PBR28, is reduced, which should result in a lower SUV. The higher SUV could perhaps be explained by lower plasma protein binding in the sampled rats and/or a higher release from peripheral tissue.

Ideally a comparison between V_T_ and SUV should also be obtained for mice. However, sampling in mice is challenging due to practical limitations. An average weight mouse has around 1.5 ml total blood and from that only a very small amount (i.e. less than 0.15 mL) could be taken due to ethical regulations on one occasion. This means that about 10 μL per sample could only be taken to have sufficient time points and it would not be possible to also obtain measurements of activity in plasma. To perform metabolite analysis as well a separate group of mice should then be included and an average metabolite curve of those should then be used during the data analysis. This can induce variations, hampering the proper comparison between the V_T_ and SUV.

When comparing the SUV of [^11^C]PBR28 between rats and mice, significantly higher SUV was found in the hypothalamus and brainstem of mice. This could be due to species differences in the regional expression of TSPO in rats and mice, but can also be attributed to the limited spatial resolution for the small animal PET system. Spill-over from regions with high uptake outside the brain could affect the SUV of brain regions. Regions which are located at the base of the skull, like hypothalamus and brainstem, are more prone to partial volume effects (PVE). This effect is larger in mice than in rats as the brain is much smaller, while the resolution of PET is unchanged. Despite of the difference in the hypothalamus and brainstem uptake between mice and rats, the distribution of SUV across brain regions is similar.

For both rats and mice the test-retest variability was good, as for most of the brain regions the test-retest variability was lower than the variability between subjects (i.e. than the relative standard deviation). For the rats the ICC values indicated moderate (2 of the 8 brain regions), substantial (3 of the 8 brain regions) or excellent (3 of the 8 brain regions) agreement, showing that in rats the reliability of the test-retest measurement was good. For the mice the ICC values indicated substantial agreement for the thalamus and a fair or slight agreement for the other brain regions, showing a poor reliability of the test-retest measurement. As the test-retest variability of the mice was good, the low ICC values are related to the larger variability between subjects.

In conclusion, we have shown that SUV can be used as an outcome measure in longitudinal [^11^C]PBR28 PET studies in healthy rodents. The next step is to determine the reliability of the SUV in rodents with neuroinflammation.

## References

[1] Doorduin J, de Vries EFJ, Dierckx RA, Klein HC. PET imaging of the peripheral benzodiazepine receptor: monitoring disease progression and therapy response in neurodegenerative disorders. Curr Pharm Des. 2008;14: 3297–3315. 19075709 10.2174/138161208786549443

[2] Venneti S, Lopresti BJ, Wiley CA. The peripheral benzodiazepine receptor (Translocator protein 18kDa) in microglia: from pathology to imaging. ProgNeurobiol. 2006; 80(6): 308–22. 17156911 10.1016/j.pneurobio.2006.10.002 PMC1849976

[3] Papadopoulos V, Baraldi M, Guilarte TR, Knudsen TB, Lacapère JJ, Lindemann P, et al Translocator protein (18kDa): new nomenclature for the peripheral-type benzodiazepine receptor based on its structure and molecular function. Trends Pharmacol Sci. 2006;27(8): 402–409. 16822554 10.1016/j.tips.2006.06.005

[4] Papadopoulos V. In search of the function of the peripheral-type benzodiazepine receptor. Endocr Res. 2004;30: 677–684. 15666811 10.1081/erc-200043971

[5] Papadopoulos V, Amri H, Li H, Boujrad N, Vidic B, Garnier M. Targeted disruption of the peripheral-type benzodiazepine receptor gene inhibits steroidogenesis in the R2C Leydig tumor cell line. J Biol Chem. 1997;272: 32129–32135. 9405411 10.1074/jbc.272.51.32129

[6] Hashimoto K, Inoue O, Suzuki K, Yamasaki T, Kojima M. Synthesis and evaluation of ^11^C-PK 11195 for in vivo study of peripheral-type benzodiazepine receptors using positron emission tomography. Ann Nucl Med. 1989;3(2): 63–71. 2561896 10.1007/BF03164587

[7] Venneti S, Lopresti BJ, Wiley CA. Molecular imaging of microglia/macrophages in the brain. Glia. 2013;61: 10–23. 10.1002/glia.22357 22615180 PMC3580157

[8] Wang M, Yoder KK, Gao M, Mock BH, Xu X-M, Saykin AJ, et al Fully automated synthesis and initial PET evaluation of [^11^C]PBR28. Bioorg Med Chem Lett. 2009;19: 5636–9. 10.1016/j.bmcl.2009.08.051 19716298 PMC2743750

[9] Imaizumi M, Kim HJ, Zoghbi SS, Briard E, Hong J, Musachio JL, et al PET imaging with [^11^C]PBR28 can localize and quantify upregulated peripheral benzodiazepine receptors associated with cerebral ischemia in rat. Neurosci Lett. 2007;411: 200–5. 17127001 10.1016/j.neulet.2006.09.093

[10] Hirvonen J, Kreisl WC, Fujita M, Dustin I, Khan O, Appel S, et al Increased in vivo expression of an inflammatory marker in temporal lobe epilepsy. J Nucl Med. 2012;53: 234–40. 10.2967/jnumed.111.091694 22238156 PMC3832892

[11] Kreisl WC, Lyoo CH, McGwier M, Snow J, Jenko KJ, Kimura N, et al In vivo radioligand binding to translocator protein correlates with severity of Alzheimer’s disease. Brain. 2013;136: 2228–38. 10.1093/brain/awt145 23775979 PMC3692038

[12] Turkheimer FE, Edison P, Pavese N, Roncaroli F, Anderson AN, Hammers A, et al Reference and target region modeling of [^11^C]-(R)-PK11195 brain studies. J Nucl Med. 2007;48(1): 158–67. 17204713

[13] Briard E, Zoghbi SS, Imaizumi M, Gourley JP, Shetty HU, Hong J, et al Synthesis and evaluation in monkey of two sensitive ^11^C-labeled aryloxyanilide ligands for imaging brain peripheral benzodiazepine receptors in vivo. J Med Chem. 2008;51(1): 17–30. 18067245 10.1021/jm0707370

[14] Nagy K, Tóth M, Major P, Patay G, Egri G, Häggkvist J. Performance evaluation of the small-animal nanoScan PET/MRI system. J Nucl Med. 2013;54: 1825–32. 10.2967/jnumed.112.119065 23990683

[15] Szanda I, Mackewn J, Patay G, Major P, Sunassee K, Mullen GE, et al National Electrical Manufacturers Association NU-4 performance evaluation of the PET component of the NanoPET/CT preclinical PET/CT scanner. J Nucl Med. 2011;52: 1741–7. 10.2967/jnumed.111.088260 21969357

[16] Logan J. Graphical analysis of PET data applied to reversible and irreversible tracers. Nucl Med Biol. 2000;27: 661–70. 11091109 10.1016/s0969-8051(00)00137-2

[17] Landis JR, Koch GG. The measurment of observer agreement for categorrical data. Biometrics. 1977;33: 159–74. 843571

[18] Fujita M, Imaizumi M, Zoghbi SS, Fujimura Y, Farris AG, Suhara T, et al Kinetic analysis in healthy humans of a novel positron emission tomography radioligand to image the peripheral benzodiazepine receptor, a potential biomarker for inflammation. Neuroimage. 2008;40(1): 43–52. 18093844 10.1016/j.neuroimage.2007.11.011 PMC2265774

[19] Yoder KK, Territo P, Hutchins GD, Hannestad J, Morris ED, Gallezot J, et al. Comparison of standardized uptake values with volume of distribution for quantification of [^11^C]PBR28, in: International Symposium on Functional Neuroreceptor Mapping of the Living Brain. 2014; p. 87.

[20] Doorduin J, Klein HC, Dierckx RA, James M, Kassiou M, de Vries EFJ. [^11^C]-DPA-713 and [^18^F]-DPA-714 as new PET tracers for TSPO: a comparison with [^11^C]-(R)-PK11195 in a rat model of herpes encephalitis. Mol Imaging Biol. 2009;11: 386–98. 10.1007/s11307-009-0211-6 19330384 PMC2763079

